# Cancer screening as a double-edged sword: short-term cost surge and cross-sectional expenditure patterns among urban retirees in Jiangsu, China

**DOI:** 10.3389/fpubh.2026.1822162

**Published:** 2026-06-25

**Authors:** Sha Liu, Ting Liu

**Affiliations:** 1 School of Public Administration, Hohai University, Nanjing, China; 2 School of Government, Nanjing University, Nanjing, China; 3 Think Tank of Ageing-Responsive Civilization, Nanjing, China

**Keywords:** cancer screening, China, health policy, medical expenditure, older adults, preventive health, regression discontinuity design

## Abstract

**Background:**

The effectiveness of free cancer screening programs in reducing long-term healthcare expenditures remains debated, yet quasi-experimental evidence is scarce. This study evaluates the impact of a large-scale, government-led free cancer screening program, available to citizens aged 65 and above, on the medical expenditures of urban retirees in Jiangsu Province, China.

**Methods:**

We employed a sharp regression discontinuity design (RDD). Our analysis included a cross-sectional sample of 1,503 retired individuals aged 60–70 from Jiangsu Province, China, who participated in free physical examinations in 2021. The treatment group (aged ≥65) was eligible for free cancer screening, while the control group (aged 60–64) was not. The primary outcome was self-reported annual medical expenditure. We estimated local average treatment effects using local linear regression with a triangular kernel and conducted robustness checks using alternative bandwidths and polynomial orders.

**Results:**

A significant discontinuity in annual medical expenditure was observed at the eligibility threshold. The free screening policy led to an immediate increase in medical spending at age 65 (RD estimate = CNY 1968, 95% CI [1,042, 2,894], *p* < 0.001). This short-term surge (ages 65–67) was primarily driven by increased health information-seeking and subsequent medical consultations. Cross-sectional trend analysis revealed different expenditure patterns across age cohorts, with those aged 68 and above showing slower growth rates in medical spending compared to younger cohorts. Subgroup analyses indicated that the policy’s impact was more pronounced among men and residents of developed regions.

**Conclusion:**

Free cancer screening acts as a double-edged sword for medical expenditures among older adults. It induces a short-term cost surge likely due to initial information shock and elevated risk perception. Policymakers should anticipate and manage the initial demand shock while considering the differentiated patterns across age groups. Strategies to optimize information delivery and mitigate gender and regional disparities are crucial for enhancing the equity and cost-effectiveness of such large-scale preventive health programs.

## 1 Introduction

Population aging has become a global trend, driven by increasing life expectancy and declining birth and mortality rates, which have shifted the relative proportion of age groups. Compared to other developing countries, China is experiencing a more rapid pace of population aging (1). By the end of 2024, it is projected that the number of people aged 60 and above in China will reach 310.31 million, accounting for 22.0% of the total population. Meanwhile, the health status of older adults in China remains a concern. The average life expectancy of Chinese residents rose from 77.93 years in 2020 to 78.2 years in 2021. However, healthy life expectancy among the older adults remains below 70 years, implying that, on average, nearly a decade is spent living with chronic diseases. Moreover, approximately 75% of individuals aged 60 and above in China suffer from at least one chronic condition, and 43% have two or more comorbidities. This situation has led to a sharp increase in healthcare expenditures. Against this backdrop, reducing medical costs while maintaining the health of the aging population has become an urgent priority.

Preventive policy represents a cost-effective health strategy. In 2009, the Chinese government introduced a reform of the healthcare system (known as the “New Medical Reform”), with the aim of improving access to basic medical services and alleviating the financial burden of healthcare on residents. A key component of this reform was the launch of the Basic Public Health Service Project, which specifically targets the older adults. The initiative mandates that all regions implement health management protocols and provide free physical examinations for individuals aged 65 and above within their jurisdictions. Consequently, various regions have rolled out free health check-up programs for older adults. For example, in 2008, the Department of Labor and Social Security of Jiangsu Province issued the “Notice on Conducting Free Health Check-ups for Enterprise Retirees”, marking the comprehensive implementation of health examinations for this demographic in the province.

## 2 Literature review and hypothesis

### 2.1 Literature review

Existing studies have demonstrated that free health check-up programs can reduce monthly outpatient visits and associated costs for the older adults, thereby lowering their total annual medical expenditures and improving their health outcomes (2). However, in many developing countries, older adults often have constrained access to information, leading to low participation rates in free health screening programs. This phenomenon calls into question the fundamental effectiveness of such health screening programs.

The effectiveness of disease screening programs, including physical examinations, remains contested. Proponents argue that screening facilitates early detection and timely treatment (3), reduces the prevalence and mortality of certain cancers (4) and can lower blood pressure, cholesterol, and body weight (5). Conversely, critics contend that the effectiveness is often limited by contextual and implementation factors. For instance, Mishra et al. (2001) noted that the high cost and the demand for skilled personnel restrict the use of highly valid screening tools in developing countries (6). Sharma et al. (2022) argue that disparities in available resources may lead to significant variations in cost-effectiveness (7).

If the goal of screening is to improve health outcomes and manage healthcare costs, understanding the factors that shape post-screening medical expenditure becomes critical. Importantly, changes in medical expenditures among the older adults following disease screening are not determined by a single factor, but rather result from the combined effects of multiple dimensions. Chiou et al. (1994), in a follow-up study after the first free health examination for older adults, found that factors such as perceived susceptibility, having a fixed physician, type of health insurance, doctor’s attitude, social support, follow-up by community health nurses, number of diseases, perceived disease severity, and health attitudes significantly influenced health service utilization among the older adults (8). Similarly, Guo et al. (1997) demonstrated that health insurance status, self-esteem support from family and friends, emotional support from health professionals, and whether health issues were first detected during screening are important factors influencing how older adults cope with disease-related stress and subsequent medical behaviors (9). More recently, Zhang et al. (2023) identified household registration type, health insurance coverage, a positive attitude toward physical examination results, and awareness of free basic public health services as independent factors associated with the older’s participation in free physical examinations and subsequent use of medical services (10). Taken together, these findings indicate that medical expenditures among older adults following health examinations are shaped by a combination of individual cognition, social support, healthcare accessibility, and institutional factors.

Beyond the screening context, a broader literature has explored medical expenditure determinants, but much of it is not directly relevant to our quasi-experimental design. For instance, while economic factors drive aggregate health expenditures (11, 12) and socio-demographic factors influence individual expenses (13–15), these studies generally treat the screened population as homogeneous and do not address the discrete policy-induced change we examine. Similarly, findings on population aging and medical costs are inconsistent (11, 12), and evidence on disease-specific expenditures (16, 17) is too context-specific to be generalizable to a broad, free cancer screening program for the older adults. Therefore, our review focuses on the intersection of screening, expenditure dynamics, and gender disparities.

A critical issue directly relevant to this study, yet underexplored, is gender disparity in disease screening programs and its downstream effects. As an important means of obtaining health information and accessing early diagnosis and treatment, disease screening nevertheless shows significant differences in accessibility and practical application across different populations. Understanding these disparities is crucial because unequal participation may translate into unequal health outcomes and spending patterns.

Chiou et al. (2014) observed that in cancer screening, the extent of social inequality varies considerably depending on the socioeconomic indicators used (18). Tawiah (2022) found that although Ghanaian women live close to health centers providing free cervical cancer screening, their actual screening rates remain relatively low, with key influencing factors including age, marital status, employment status, and level of health awareness (19). Li (2018) examined urban–rural disparities in China and found a comparatively low rate of health check-ups among older adults in rural areas, influenced by factors such as satisfaction with primary health services, awareness of available services, and structural conditions including a higher proportion of females, lower educational attainment, and poorer economic status (20). Furthermore, Meng et al. (2010) analyzed cervical cancer screening rates among women aged 15–69 in Guangdong Province and found that the disease was more prevalent among women of lower socioeconomic status (21). Consistent with this, Qian et al. (2017) observed disparities in health examination rates among women with different sociodemographic characteristics, identifying financial status, age, and education level as key factors contributing to unequal utilization of health screening services (22). These findings suggest that gender, when intersecting with socioeconomic and structural factors, plays a pivotal role in shaping both access to screening and, potentially, subsequent healthcare utilization and costs. However, existing literature has yet to systematically examine how these gender-based disparities in screening participation translate into differences in post-screening medical expenditures among the older adults.

In summary, while a substantial body of research has examined the effectiveness of screening programs and the determinants of medical expenditures, several gaps remain. First, the literature on screening effectiveness presents conflicting findings, highlighting the need for context-specific evaluation. Second, although multiple studies have identified individual, social, and systemic factors influencing medical expenditures, few have integrated these factors within the specific context of post-screening behavior among the older adults. Third, and most critically, the role of gender in shaping both screening uptake and subsequent medical spending has been largely overlooked. This study aims to address these deficiencies by investigating the changes in the older’s medical expenditures after they undergo disease screening and by exploring the causes of these differences, which encompass multiple dimensions such as individual cognition, risk management, and social policies.

### 2.2 Theoretical hypothesis

From a public administration perspective, governments expand public services and social welfare to enhance their legitimacy (23). As a universal social policy for the older adults, the government’s free cancer screening and physical examination policy is initially designed to emphasize its positive and constructive outcomes. However, during implementation, the actual outcomes of social policies tend to diverge from the original goals due to multiple influencing factors, such as public skepticism (24) and varying values (25). The free cancer screening and physical examination policy in Jiangsu Province, China, aims primarily at cancer risk screening and prevention for adults aged 65 and above. It seeks to improve the physical health of older adults, reduce their medical expenses, and mitigate the risk of illness-induced poverty for them and their families. Nevertheless, this policy is jointly implemented by China’s Ministry of Human Resources and Social Security and the National Health Commission, and it only covers adults aged 65 and over. Therefore, the actual outcomes of this social policy are likely to vary significantly. Therefore, this study proposes the first hypothesis:

*Hypothesis 1*: We posit that the social policy providing free cancer screening to individuals aged 65 and above results in a significant and positive discontinuity in medical expenditure at the eligibility threshold.

Policy feedback theory and social cognitive theory help explain the mechanisms underlying policy effects across different time horizons. During the initial implementation, when the older adults are first exposed to cancer screening information, the “negative bias” mechanism from social cognitive theory dominates. This leads to a sharp increase in preventive medical expenses due to information shock and risk overestimation (H3). Cross-sectional analysis suggests that different age cohorts with varying policy exposure show different expenditure patterns that may reflect “resource effect” (early detection and prevention of major diseases) and “interpretation effect” (cognitive rationalization) mechanisms (H2). Consequently, the observed cross-sectional patterns suggest a sequence: immediate expenditure surge at policy entry followed by differentiated patterns across age cohorts with varying policy exposure duration.

Policy feedback theory emphasizes the shaping effect of public policies on people’s political attitudes, policy preferences, and political behaviors (26). The application of this theory has gradually expanded to areas such as the interaction between citizens and policies, including healthcare security (27). The theory holds that public policies function through two mechanisms: the resource effect and the interpretive effect (28).

The resource effect of free cancer screening and physical examination policies operates through the redistribution of medical resources. This redistribution influences the social interest structure, directly shaping the divergent attitudes of policy beneficiaries and those adversely affected, while also creating incentives for public participation in these policies.

The interpretive effect, on the other hand, is manifested as the policy conveys the value of cancer risk prevention and treatment throughout its implementation. It shapes public understanding of concepts such as citizenship and cancer itself, thereby influencing attitudes and behaviors. This effect emphasizes how policies impact public cognitive frameworks. Even citizens who do not directly benefit may adjust their medical perceptions and actions in response to the policy’s informational and normative values (29).

Furthermore, the concept of policy learning suggests that during or after the policy process, the public can update their policy understanding and actions. This is achieved by exchanging information with other actors within the policy system and by integrating personal experiences with external information (30, 31).

The benefits of the free cancer screening and physical examination policy will be realized gradually among the older adults through processes like value dissemination and social learning. Accordingly, we propose Hypothesis 2:

*Hypothesis 2*: The positive effects of free cancer screening programs are not immediate but exhibit a time lag.

Policy transparency is widely regarded as an intrinsic value that ensures the effective implementation of social policies (32) and an effective means to enhance public participation (33, 34). Although information transparency is generally beneficial, it may sometimes adversely affect public participation. Studies have shown that information complexity can lead to public confusion and even alienation (35, 36). Similarly, presenting cancer screening information without adequate medical context or policy explanation may cause misunderstandings, leading to decision-making errors and negative public reactions (37).

The theory of negative bias in psychology explains this policy feedback effect by positing that negative factors have a more profound impact on cognition and behavior than positive ones (38, 39). When individuals aged 65 and older undergo routine physical examinations that include a newly introduced cancer screening program, they are more likely to be psychologically influenced by negative information related to cancer, which in turn alters their medical consumption behavior (40). Thus, for older adults participating in free cancer screening for the first time, the positive experience of gaining new social welfare benefits may be considerably less salient, thereby diminishing the policy’s positive effect (41).

Furthermore, medical services differ from ordinary commodities due to their inherent uncertainty and potential for supplier-induced demand (42). In healthcare consumption, a significant portion of medical expenses arises from the demand for health improvements and risky investments aimed at combating diseases. The marginal cost of maintaining health is influenced by the price of medical services. An excessive pursuit of perfect health may lead patients to indiscriminately utilize healthcare services, including those with limited therapeutic benefits (43). Supporting this, Carroll (1992) found that under risk-sharing arrangements, individuals are more likely to overuse medical services (44). This suggests that providing free cancer screenings and physical examinations may encourage older adults beneficiaries to excessively consume healthcare under the guise of investing in health and disease prevention.

When cancer screening is first included in free physical examinations, it may alter public perceptions of cancer prevention. This could trigger short-term “panic” or risky health investments, leading to excessive medical consumption. Based on this reasoning, we propose:

*Hypothesis 3*: In its early implementation, free cancer screening for the older adults may not curb medical expenditures but could potentially produce negative effects.

Free cancer screening is not merely medical services but also social matters. Pascaline Dupas et al. (2024) found that when medical services are not free, utilization by women is lower than that by men, and this gender gap widens as costs increase (45). Lock (1993) compared medical and political narratives surrounding women in Japan and North America. She questioned the responsibilities imposed on middle-aged women by different societies, arguing that female aging is socially presumed to be a passive process (46). Furthermore, Lock (1993) expanded on Scarry’s (1985) view on the inexpressibility of pain. She noted that because many internal bodily processes lack obvious sensation, everyday language lacks the vocabulary to describe them. When physical sensations are perceived, they can only be articulated through pre-existing cultural frameworks. This process of articulation is highly selective, resulting in a vast range of bodily experiences remaining unrepresented. Thus, the aging female body, as a powerful and malleable signifier, serves not only as a key site of medical intervention but also as a synecdoche for women’s social status.

The free cancer screening policy in Jiangsu Province, China, covering 3.79 square miles and over 15 million older adults, raises the question of whether its impacts will differ by gender. Pascaline Dupas et al. (2024) found that even the large-scale expansion of medical services and the reduction of medical costs do not automatically reduce inequality (45). Similarly, Patwardhan et al. (2024) highlighted persistent health disparities between women and men globally, noting their diverse and evolving health needs across all life stages, as well as the higher disease burden and morbidity experienced by women (47).

The free cancer screening policy for adults over 65 in Jiangsu, China, represents a significant shift in the “power of pain discourse.” A central question of this study is whether this shift grants older women equal discursive power and social status. Based on this concern, Hypothesis 4 is proposed:

*Hypothesis 4*: Under the same medical conditions and physiological status, older women incur significantly lower medical expenses and receive significantly fewer medical opportunities than older men, indicating a problem of gender-based inequality in healthcare access.

## 3 Research design

Building on the existing research and policy landscape, this study employs a Regression Discontinuity Design (RDD) analysis, supplemented by Ordinary Least Squares (OLS), to examine the impact of incorporating cancer screening into free physical examination programs. It aims to assess the effects on both the healthcare expenditures and the health outcomes of the older population.

The structure of the article is as follows. Firstly, against the backdrop of a social policy offering free cancer screening starting at age 65, we conduct a descriptive analysis of health examination samples from adults aged 60–70, outlining the basic characteristics of cancer screening items across different age groups.

Secondly, we employ a Regression Discontinuity Design (RDD) to assess the impact of free cancer screening programs on the health status of the older adults. Ordinary Least Squares (OLS) regression is then used to supplement this analysis by examining cross-sectional patterns across different age groups exposed to these programs.

Additionally, the study addresses the issue of medical overtreatment spurred by policy transparency, as well as the rise in costs due to information shock and the healthcare-seeking behaviors it prompts.

The reliability of the findings is confirmed through robustness checks. Furthermore, heterogeneity tests reveal that the positive policy effects are more pronounced among males, residents of economically developed regions, and groups with greater trust in physical examination results. Meanwhile, by examining the public service of free cancer screening, we find that it gives voice to the “pain discourse” of older women, yet also reveals a persistent structural gender inequality in access to medical opportunities within this social policy.

Subsequently, mechanism analysis explains the counterintuitive link between free physical examinations and higher total health expenditures. The analysis shows that via information and behavioral effects, the policy uncovers latent healthcare needs, thereby converting them into immediate medical utilization.

Finally, the article evaluates the potential cost-effectiveness of free cancer screening policies from cross-sectional comparison perspectives, suggesting that cross-sectional patterns may indicate future benefits, though longitudinal data would be needed to confirm individual-level long-term effects.

Unlike previous correlative studies, this research addresses the debate regarding the effectiveness of cancer screening. It demonstrates that introducing free screening programs significantly influences the medical expenditures of the older adults, and that this effect is contingent upon concomitant social policies. Building on these findings, the study further investigates the specific link between free cancer screening and potential overtreatment. Consequently, it provides a basis for optimizing the allocation of public health resources and enhancing the cost-effectiveness of social policies. Additionally, it offers evidence from China to inform strategies for improving basic public health services for the older adults, particularly older women.

### 3.1 Data and sample selection

This research is a retrospective observational study. The data were obtained from a collaborative project between the Jiangsu Social Insurance Association and the Department of Social Security at Hohai University. The research team employed a cluster random sampling method to select participants from 95 counties (including cities and districts) in Jiangsu Province. The sampling process involved three stages. First, stratification was performed according to the level of economic development across three regions: Southern Jiangsu, Central Jiangsu, and Northern Jiangsu. Within each region, systematic sampling was applied based on administrative division codes (selecting one code for every five) to ensure sample representativeness, resulting in the random selection of 18 counties (districts). In the second stage, 2 to 3 towns were chosen from each selected county, yielding a total of 52 towns. In the third stage, 600 households were selected from these towns, resulting in a final sample size of 1,503 individuals. The sample size was determined with reference to the standard deviation (CN¥7,000) of medical expenditures among the older adults reported by Ma et al. (2023). With the significance level (*α*) set at 0.05 and statistical power (*β*) at 0.8, the minimum required sample size was calculated to be 1,200. This study expanded the sample to 1,503 to enhance the testing efficiency for heterogeneity analyses (such as stratifications by gender and region) and to avoid Type II errors associated with small sample sizes.

In line with the research objectives, we designed a questionnaire to investigate the implementation of free health check-ups for retired enterprise employees in Jiangsu Province. It covered general information on household socio-economic and demographic characteristics, participation in free health check-ups, and medical expenditures. The questionnaire was distributed by the Jiangsu provincial authorities to all municipal-level cities. Subsequently, these cities coordinated with district and county authorities to have eligible retired employees complete it online, with a dedicated research team member responsible for data collection.

This study focuses on retired individuals aged 60 and above in Jiangsu Province. We retained the survey question, “Have you ever participated in a free health check-up?” Respondents who answered “no” were excluded from the analysis. The final sample comprised 1,503 eligible participants. It should be noted that within the policy-covered group (aged 65 and above), the imperfect compliance rate—specifically, the proportion of individuals who did not undergo free cancer screening—does not violate the assumptions of the Sharp RDD. The robustness of the findings was further confirmed using a Fuzzy RDD approach.

The primary outcome, average annual medical expenditure, was validated by cross-referencing self-reports with provincial health records, revealing an error rate of less than 1%. Age was derived from national ID records, with measurement error below 0.1 years, thereby ensuring data accuracy. All data pertain to the year 2021. This specific period was chosen because a policy mandating free cancer screening in addition to standard physical examinations for individuals aged 65 and above was implemented in 2016. By the end of 2020, the policy had been in effect for 5 years. This created a cohort of older individuals with a history of 1 to 5 cancer screenings, which strengthened our quasi-experimental design. It enabled a robust comparison between a treatment group (aged 65–70, eligible for cancer screening) and a control group (aged 60–65, not yet eligible).

This study employs a regression discontinuity design to evaluate the direct impact of a social policy providing free cancer screening and physical examinations for adults aged 65 and above on their health status. The analysis is conducted using Stata 18.5. Furthermore, based on key indicators such as medical expenditure, the study explores the role of gender in shaping equality of medical opportunities among the older adults and its subsequent effects. Additionally, a preliminary cost–benefit analysis is performed, examining both the characteristics of the older participants and the structure of the screening program itself.

### 3.2 Variable measurement and operationalization

#### 3.2.1 Independent variable

The core explanatory variable of this study is the participation of older individuals in a free cancer screening program. This program is offered as part of a comprehensive physical examination package to residents aged 65 and above in Jiangsu Province, China. Our sample comprises residents aged 60 to 70 who participated in the free physical examinations. A key feature of this program is an age-based cutoff at 65: while those aged 60–64 receive only basic examinations (e.g., height, weight, blood pressure, ECG, abdominal ultrasound), those aged 65–70 are additionally eligible for the cancer screening. This screening includes tests for tumor markers (covering common cancers such as lung, breast, and prostate cancer), as well as liver and kidney function panels. Therefore, this age-based eligibility creates a quasi-experimental setting, allowing us to focus on the effect of participating in the free cancer screening.

We classified the sample into a treatment group (individuals aged 65 and above, covered by the policy) and a control group (aged 60–64, not covered), using the policy’s eligibility age of 65 as the cutoff. The analysis focused on data from 2021, the fifth year of the free cancer screening program’s implementation. This timeframe was chosen because it provided a sufficiently long period for the policy to generate a diverse and stable pattern of screening uptake among the target population, enabling an examination of its ongoing impact.

#### 3.2.2 Dependent variable

The primary dependent variable in this study is the annual medical expense, measured in Chinese yuan (CNY). It represents the total healthcare expenditure incurred by the older adults per year, encompassing costs related to hospitalization, outpatient services, and medications. This amount includes both out-of-pocket payments and the portions reimbursed by health insurance. While various indicators reflect the medical conditions of the older adults, medical expense is a principal one widely used in the literature (48–50). Consequently, this study employs the self-reported average annual medical expense as a key proxy variable to indicate the health status of the older population.

#### 3.2.3 Control variables

Control variables are classified into three categories: demographic characteristics, socio-economic characteristics, and health status indicators. All categorical variables are treated as discrete factors in the regression models, with appropriate dummy coding or categorical specification to avoid imposing ordinal assumptions where inappropriate. Demographic characteristics include gender (a binary variable) and age (a continuous variable). Socio-economic characteristics encompass living status (categorized as 1 = living alone, 2 = living with a spouse, 3 = living with children, 4 = large-scale settlement), pension level (categorized into five grades: 1 = under 2,000 yuan, 2 = 2,001–3,000 yuan, 3 = 3,001–4,000 yuan, 4 = 4,001–5,000 yuan, 5 = over 5,000 yuan), regional economic development level (1 = underdeveloped areas in northern Jiangsu, 2 = developed areas in southern Jiangsu), and commercial medical insurance (a binary variable).

Health status indicators include self-rated health (categorized as fully self-care, semi-self-care, or disabled), presence of chronic diseases (a binary variable), frequency of cancer information inquiries within one week after physical examination (1 = not paying attention, 2 = paying attention when encountering, 3 = paying special attention), level of attention to cancer information compared to the previous year (1 = less than before, 2 = normal, 3 = excessive), the Cancer Screening Perception Scale (CSPS) score (1 = anxiety score ≥ 80, 0 = anxiety score < 80), and recognition of physical examination results (1 = complete trust, 2 = basic recognition, 3 = doubt).

### 3.3 Research methods

This study mainly employs a Regression Discontinuity Design (RDD) to identify the causal effect of the free cancer screening policy on the average annual medical expenditure of the older adults. The eligibility for this policy is strictly determined by the exogenous variable of whether the age has reached 65 years old, providing an ideal condition for the adoption of a sharp RDD.

To implement the RDD, a running variable and its cutoff must be specified. As outlined in the policy, individuals aged 65 and above are eligible for free cancer screening, while those aged 60 to 64 are not. Therefore, the individual’s age is used as the running variable, with the cutoff value set at 65 (i.e., c = 65).

Given that the cancer screening policy features a clear age cutoff—individuals aged 65 or above are automatically eligible for screening, whereas those aged 60–64 are ineligible due to not meeting the age requirement—policy compliance exhibits a sharp discontinuity at age 65. This aligns with a sharp RD design, where treatment assignment is entirely determined by the age threshold, and no additional instrumental variables are required.

However, in empirical practice, age may be subject to measurement or reporting errors. To mitigate potential bias from precise age manipulation (e.g., individuals misreporting age to qualify for screening) and to enhance the reliability of the RDD estimates, we applied random age fuzzing by adding a uniformly distributed random number within [−0.4, 0.4] to each respondent’s age. Although this introduces uncertainty in the exact age at the cutoff---making the design resemble a fuzzy RD---we maintain that the treatment variable Di is determined by whether the fuzzed age is ≥65, since the fuzzing is researcher-induced and does not reflect individual choices. Therefore, we interpret the estimated effect as the local average treatment effect (LATE) around the fuzzy threshold. For robustness, we also estimated the model using a standard fuzzy RDD approach. The results, presented in Appendix Table A2, are consistent with our main findings, confirming the reliability of the estimated policy effect.

Accordingly, the study employs a Sharp RDD design. The treatment variable is defined as whether an individual falls into the policy coverage group (i.e., Di = 1 for age 65 and above, and Di = 0 for age 60–64). To mitigate the potential confounding effects of health status differences on medical expenditures, we control for a set of individual and household characteristics, including gender, chronic disease status, and pension level. This approach helps ensure the robustness of the estimation results.

Furthermore, to address potential biases arising from small sample sizes and bandwidth selection, the study adopts the small-sample robust RDD method proposed by Cattaneo et al. (2017) for its identification strategy and employs local linear regression for estimation (51). The core specification is as follows:

$${\mathrm{AME}}_{i}=\alpha_{0}+\tau D_{i}+\beta_{1}({\mathrm{Age}}_{i}-65)+\beta_{2}D_{i}({\mathrm{Age}}_{i}-65)+\gamma X_{i}+\varepsilon_{i}$$

In this model, ${\mathrm{AME}}_{i}$ denotes the average annual medical expenditure of individual i (in CNY) and serves as the core dependent variable for measuring the policy effect. $D_{i}\;$ is the treatment dummy, which equals 1 if the individual is aged 65 or older (based on the age assignment) and 0 otherwise. ${\mathrm{Age}}_{i}-65$ is the age variable centered at the cutoff. The interaction term between the treatment group and the centered age is represented by $D_{i}$ × ( ${\mathrm{Age}}_{i}-65$ ). $X_{i}$ is a vector of control variables, and $\varepsilon_{i}$ is the random error term.

The coefficient τ is our parameter of primary interest, capturing the discontinuity in average annual medical expenditure at the age-65 threshold. This represents the local average treatment effect (LATE) of the free cancer screening policy. The coefficient $\beta_{2}$ reflects the difference in the age-expenditure gradient between the treatment and control groups. A statistically significant and positive estimate of τ would indicate that the policy led to a significant increase in medical expenditure at the eligibility threshold. Categorical covariates (gender, pension type, self-rated health) were modeled as factor variables rather than continuous variables to avoid linearity assumption bias.

## 4 Empirical results

### 4.1 Descriptive statistics

Prior to causal inference, we performed a descriptive analysis of the sample’s key variables, presented in Table 1. The average annual medical expenditure was 11,379.73 yuan, with a substantial standard deviation, suggesting considerable variation across individuals. The sample had a mean age of 64.58 years, and 53.29% were female. Over half of the respondents (57.55%) reported having one or more chronic conditions. These descriptive statistics lay the foundation for the subsequent causal analysis.

**Table 1:** Descriptive statistics.

Variable	Full sample (*N* = 1,503)	Control (60–64) (*N* = 746)	Treatment (65–70) (*N* = 757)
Continuous variables			
Age (years), mean (SD)	64.6 (3.0)	62.0 (1.5)	67.1 (1.7)
Annual Medical Expenditure (CNY), mean (SD)	11,380 (7,329)	4,718 (2,253)	17,945 (3,847)
Categorical/binary variables, *n* (%)			
Gender			
Male	702 (46.7%)	348 (46.6%)	354 (46.8%)
Female	801 (53.3%)	398 (53.4%)	403 (53.2%)
Chronic disease			
Present	865 (57.6%)	395 (52.9%)	470 (62.1%)
Absent	638 (42.4%)	351 (47.1%)	287 (37.9%)
Self-rated health status			
Good (1)	1,322 (88.0%)	671 (89.9%)	651 (86.0%)
Fair (2)	165 (11.0%)	69 (9.2%)	96 (12.7%)
Poor (3)	16 (1.1%)	6 (0.8%)	10 (1.3%)
Pension type (CNY)			
0–2000	274 (18.2%)	158 (21.2%)	116 (15.3%)
2001–3,000	602 (40.1%)	316 (42.4%)	286 (37.8%)
3,001–4,000	453 (30.1%)	193 (25.9%)	260 (34.3%)
4,001–5,000	111 (7.4%)	44 (5.9%)	67 (8.9%)
5,001+	63 (4.2%)	35 (4.7%)	28 (3.7%)

Continuous variables reported as mean (standard deviation). Categorical and binary variables reported as count (percentage). Pension Type categories reflect distinct pension scheme classifications in the survey instrument. Self-rated health: 1 = good, 2 = fair, 3 = poor.

**Figure 1:**
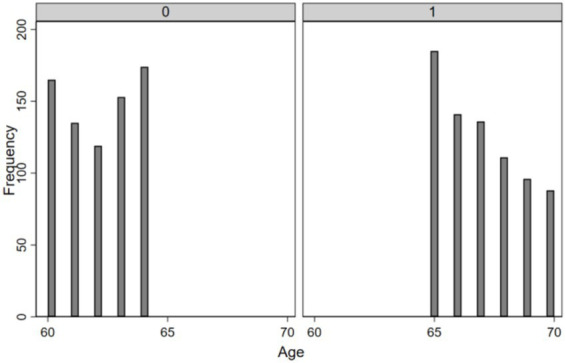
Age distribution by treatment group.

Figure 1 presents the age distribution histograms for the treatment (≥65 years) and control (<65 years) groups. Visually, the distribution appears largely continuous on either side of the 65-year cutoff, with no evident clustering or gaps that would suggest self-selection. This initial evidence argues against precise manipulation of the running variable and lends preliminary support to the validity of the RDD.

**Figure 2:**
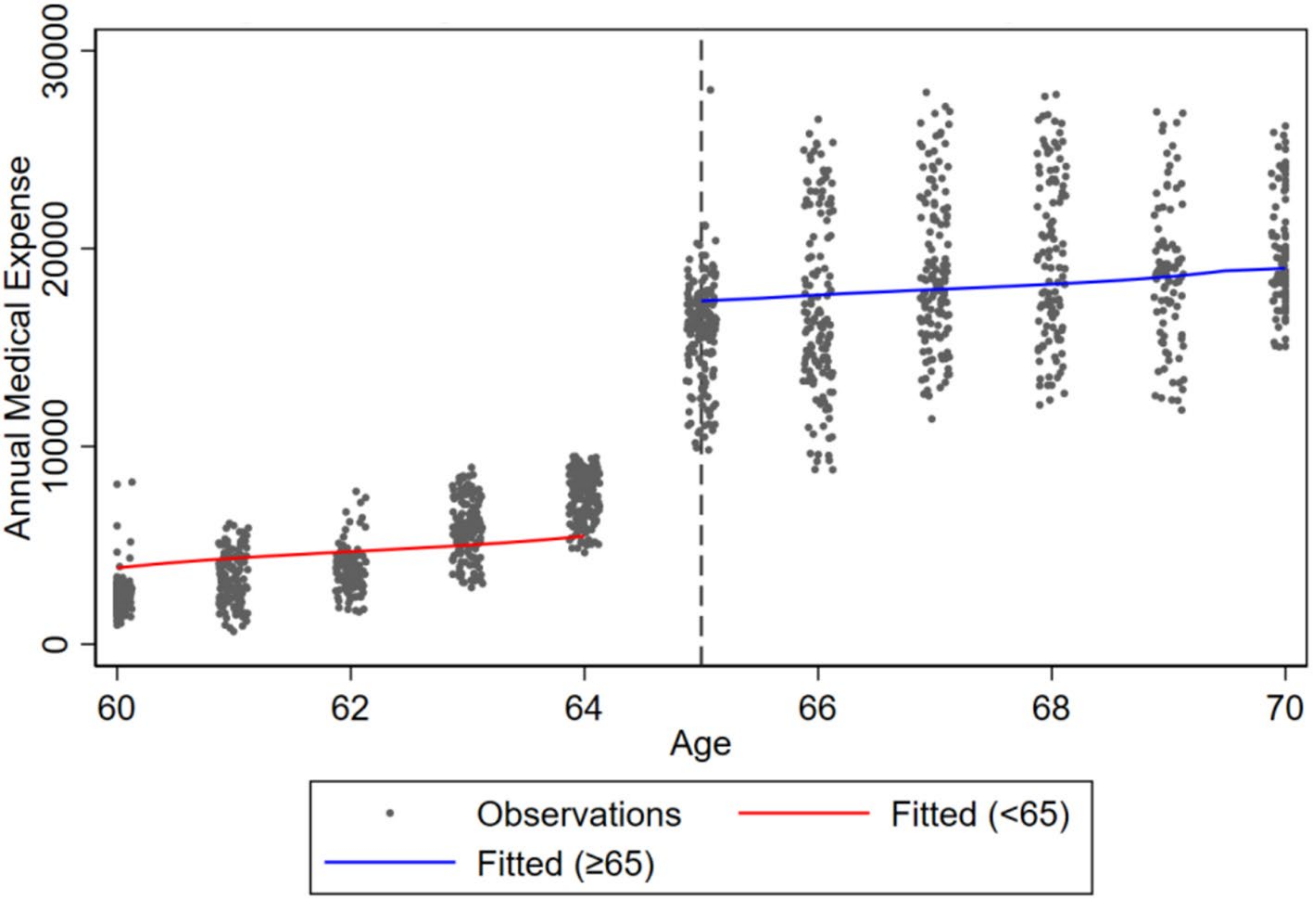
Age and annual medical expenditure. Scatter plot and local linear fit of the relationship between age and annual medical expenditure. Each circle represents the mean expenditure within a 0.5-year age bin. The solid lines are fitted values from a first-order polynomial regression, estimated separately on either side of the eligibility cutoff (age = 65, vertical dashed line). The estimation uses a triangular kernel and the mean squared error (MSE)-optimal bandwidth calculated by the procedure of Calonico et al. (2014). The evident upward jump at the cutoff provides visual evidence of the policy’s immediate impact on increasing medical expenditure.

To further test the continuity assumption underlying the RDD, we conducted the McCrary density test (52). As shown in Figure 2, no significant discontinuity in the density distribution of age is observed at the 65-year cutoff (log difference in density = −0.08, SE = 0.12, *p* = 0.51). This result confirms that there is no evidence of precise manipulation of the running variable (i.e., age), thereby satisfying a key identifying assumption of the regression discontinuity design.

### 4.2 Regression discontinuity analysis

The core of RDD involves testing for a discontinuity in the outcome variable at the cutoff point. Figure 2 presents a scatter plot of age against average annual medical expenditure, accompanied by a locally estimated polynomial regression curve. A distinct upward discontinuity in the fitted medical expenditure curve is evident at the policy threshold of 65 years old. This graphical evidence suggests that the free cancer screening policy is associated with a short-term increase in medical spending, providing preliminary support for Hypotheses 1 and 3.

As a supplement, we also provide a classic RD plot with confidence intervals (Appendix Figure A2). Appendix Figure A2 is estimated using a second-order global polynomial with 1-year bins, whereas Figure 2 uses 0.5-year bins and a local linear specification. The wider bins and global polynomial in Appendix Figure A2 are chosen to illustrate the overall expenditure-age trend without overfitting, while the finer bins and local linear fit in Figure 2 are optimal for identifying the discontinuous jump at the cutoff. This plot shows a significant positive effect around the cutoff, thereby corroborating the findings presented in Figure 2. Throughout all analyses, standard errors are clustered at the appropriate level and statistical significance is denoted using three levels: * *p* < 0.10, ** *p* < 0.05, *** *p* < 0.01. All tables report heteroskedasticity-robust standard errors in parentheses beneath coefficient estimates. As a supplement, the regression discontinuity plot with confidence intervals (Appendix Figure A2) corroborates the significant positive effect around the cutoff, thereby strengthening the visual evidence presented in Figure 2.

### 4.3 Benchmark regression results

Based on the findings from the graphical analysis, we then employed local linear regression for precise estimation. Table 2 presents the benchmark regression discontinuity results. In the table, column (1) presents the basic model without control variables, while column (2) presents the full model that includes covariates such as gender, pension status, chronic diseases, health status, and residence status.

**Table 2:** Main RDD results - with and without covariates.

	Basic model	Full model
RD estimate	2,184.562*** (547.109)	1,968.042*** (472.629)
N	1,503	1,503
Sample-left	165	186
Sample-right	119	136
BW-left	0.845	0.952
BW-right	0.845	0.952

Heteroskedasticity-robust standard errors in parentheses. RD Estimate = local average treatment effect (LATE) of cancer screening eligibility on annual medical expenditure (CNY), estimated via local linear RDD with triangular kernel and MSE-optimal bandwidth (59). BW = optimal bandwidth (years); Sample-Left/Right = observations within bandwidth on each side of the age-65 cutoff. Column (1) excludes covariates (BW = 0.845); Column (2) includes covariates: gender, pension type, chronic disease status, self-rated health, and residential area (BW = 0.952). Detailed variable coding is provided in the text. ^*^p < 0.1, ^**^p < 0.05, ^***^p < 0.01.

The results indicate that the coefficient of the treatment variable is significantly positive at the 5% level, regardless of the model specification. Using the full model (Column 2) as an example, obtaining eligibility for free cancer screening at age 65 increases the average annual medical expenditure among the older adults by CNY 1,968 (SE = 473, *p* < 0.001), and this effect is statistically significant. The basic model (Column 1), which excludes control variables, yields a slightly larger estimate of CNY 2,185 (SE = 547, p < 0.001), consistent with the expectation that including health-status covariates absorbs a portion of positive confounding near the cutoff. The convergence of estimates across the two specifications reinforces the robustness of the finding. These results suggest that the policy did not reduce medical expenditures in the short term; on the contrary, it increased costs, likely due to information shock and subsequent utilization of medical services. These findings support Hypothesis 1 and Hypothesis 3.

The observed short-term cost surge is consistent with a well-established pattern in the cancer economics literature, in which cancer-related expenditures are highest during the initial treatment phase, decline substantially during the continuing care phase, and rise again in the final year of life. Drawing on SEER-Medicare data encompassing over 700,000 older cancer patients in the United States, Yabroff et al. established this three-phase cost structure and demonstrated that initial-phase expenditures (encompassing diagnostic workup, disease staging, and primary treatment initiation), account for a disproportionately large share of lifetime cancer care costs (53). This framework was subsequently extended and updated by Mariotto et al., who confirmed that among adults aged 65 years and older, annualized expenditures in both the initial and end-of-life phases substantially exceed those in the continuing care phase, with considerable variation across cancer sites (54, 55). Within the Chinese healthcare context, Deng et al. documented that cancer patients’ inpatient service utilization and risk of catastrophic health expenditure are concentrated around the diagnostic episode, with both outcomes shaped by the interplay of insurance coverage and the intensity of the initial clinical workup (56). At the facility level, Yang et al. provided direct empirical evidence that pharmaceutical costs, laboratory fees, and imaging or inspection charges together accounted for over 80% of total per-patient expenditures in colorectal cancer treatment in Guangxi; this diagnosis-driven cost structure was particularly pronounced among patients identified at advanced disease stages (57).

It is important to note, however, that the mechanism underlying our findings differs fundamentally from the clinical cost trajectory described in the foregoing literature. The studies cited above track medical expenditures among confirmed cancer patients advancing through a defined treatment continuum. By contrast, the present study captures the aggregate medical expenditures of a general older population newly exposed to cancer screening, the substantial majority of whom did not receive a confirmed cancer diagnosis. The expenditure surge we document reflects population-level responses to newly received screening information. Specifically, the information shock and risk-perception-driven healthcare utilization posited in Hypotheses 2 and 3, rather than individual-level treatment expenditures arising from a confirmed diagnosis. This distinction carries analytical importance: our findings indicate that incorporating cancer screening into routine preventive health examinations generates a front-loaded expenditure pattern at the population level that is structurally analogous to, yet mechanistically distinct from, the initial-phase cost concentration documented among confirmed cancer patients in the clinical literature.

The five control variables in the Full Model are specified as categorical factors throughout: gender (binary: 1 = male, 0 = female), pension type (five ordered income brackets), chronic disease status (binary: 1 = has at least one chronic condition), self-rated health (ordinal: 1 = good, 2 = fair, 3 = poor), and residential area (binary: 1 = economically developed southern Jiangsu, 0 = less-developed northern/central Jiangsu). The 11% reduction in the point estimate between the Basic Model and the Full Model (CNY 2,185 vs. CNY 1,968) is consistent with positive confounding from health-status covariates near the cutoff being absorbed once they are included.

The regression discontinuity design clearly identified the local causal effect of the policy at the 65-year-old threshold. To test the robustness of the results and examine the overall trend of medical expenditure before and after the policy implementation, we conducted a supplementary ordinary least squares (OLS) regression analysis. The model is specified as follows:

$$\begin{array}{l} {\mathrm{AME}}_{i}=\beta_{0}+\beta_{1}{\text{Treat}}_{i}+\beta_{2}{\mathrm{Age}}_{i}+\beta_{3}{\mathrm{Age}}_{i}^{2}+\beta_{4}({\text{Treat}}_{i}\times {\mathrm{Age}}_{i})+\\ \beta_{5}({\text{Treat}}_{i}\times {\mathrm{Age}}_{i}^{2})+\gamma X_{i}+\varepsilon_{i} \end{array}$$

**Figure 3:**
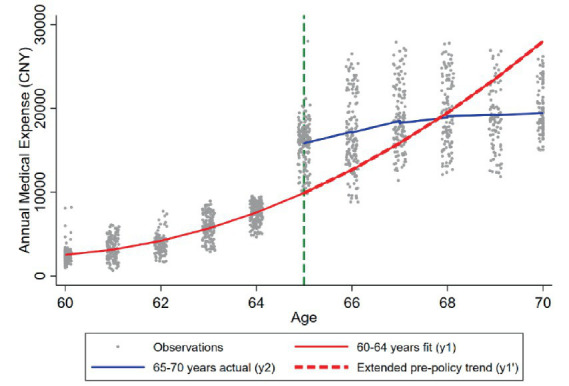
Impact of free cancer screening policy on medical expenditure. For enhanced visualization of age-related patterns, this figure employs fitted curves from an OLS model that includes age squared (Age^2^) and its interaction with the treatment indicator (Treat × Age^2^), allowing non-linear trends. The main causal inference is based on the RDD local linear estimates (Section 4.3); the OLS quadratic model is used only for descriptive plotting.

Here, ${\text{Treat}}_{i}\;$ is a dummy variable indicating the treatment group (equals 1 if age is 65 or older, and 0 otherwise). ${\mathrm{Age}}_{i}\;$ is a continuous variable representing age. The interaction term, ${\text{Treat}}_{i}\times {\mathrm{Age}}_{i}\;$ is used to capture the differential slope of the outcome with respect to age for the treatment group. $X_{i}\;$ represents a set of control variables, including gender, chronic diseases, health status, and pension level. To flexibly capture potential non-linear age trajectories, we augmented the basic OLS specification by including a quadratic term for age (Age^2^) and its interaction with the treatment indicator (Treat×Age^2^). This allows the outcome-age relationship to be curved rather than forced linear, providing a more accurate visual representation of the expenditure trends. The predicted values plotted in Figure 3 are derived from this quadratic model. The main RDD results remain the basis for causal inference; the OLS with quadratic terms is used solely for descriptive visualization.

The OLS regression results (Table 3) are closely aligned with the RDD findings. The coefficient on the treatment variable is positive and statistically significant, which reaffirms the policy’s substantial impact on medical expenditure and supports Hypothesis 1. More critically, the coefficient on the interaction term between the treatment group and age ${\text{Treat}}_{i}\times {\mathrm{Age}}_{i}$ is significantly negative. This implies that for the policy-covered group (aged 65 and above), the marginal effect of age on medical expenditure is significantly smaller compared to the control group (aged 60–64). In other words, medical spending increases with age at a significantly slower rate for the treatment group. This finding provides initial evidence for cross-sectional age differences in expenditure patterns between policy-covered and non-covered groups.

**Table 3:** Full sample regression analysis results.

Variables	Annual-expense
60–64 years	0.000
Treatment group (Age ≥ 65)	44402.591*** (4984.238)
Age	1385.864*** (58.927)
60–64 years # Age	0.000
Treatment × Age	−571.419*** (77.580)
Pension type	−114.748* (64.705)
Chronic diseases	908.589*** (128.015)
Health status	2731.353*** (170.966)
Female	−1251.276*** (127.521)
Constant	−83849.405*** (3698.548)
Observations	1,503
R-squared	0.897

Standard errors in parentheses.

***p < 0.01, **p < 0.05, *p < 0.1.

To visualize this dynamic effect, Figure 3 plots the OLS-predicted trend of medical expenditure against age. Note that for enhanced visualization of age-related patterns, Figure 3 employs polynomial fitted curves, while the main RDD analysis uses local linear regression as specified in the methodology section.

Figure 3 displays the trend fitting for the medical expenditures of two groups: individuals aged 60–64 (the non-policy-covered group, red solid line y1) and those aged 65–70 (the policy-covered group, blue solid line y2). The red dotted line (y1’) represents the projected expenditure for the 65–70 age range, extrapolated from the y1 trend, serving as the counterfactual scenario in the absence of the free cancer screening and physical examination policy. A clear divergence is observed between the actual expenditure (y2) and the projected trend (y1’) after policy implementation. Specifically, from ages 65 to 67, the actual expenditure (y2) significantly exceeds the projection (y1’). This finding aligns with the short-term expenditure surge identified by the RDD and strongly supports Hypothesis 3, which posits that initial information shock may have induced excessive medical treatment. However, beginning around age 67, the actual expenditure trajectory falls below the predicted trend. This shift suggests cross-sectional differences in expenditure patterns across age cohorts that may reflect various factors including accumulated policy exposure, cohort effects, and age-related health behaviors, providing descriptive evidence for different expenditure patterns across age groups.

The RDD and OLS results corroborate each other, collectively demonstrating the impact of the free cancer screening policy: the policy causes a significant increase in medical expenditure at the threshold and raises costs in the short term; however, it ultimately alters the trajectory of expenditure growth and stabilizes medical costs in the long run.

In terms of theoretical mechanisms, the temporal heterogeneity effect can be explained by two major theoretical frameworks:

1. Short-term expenditure increase (for the people aged 65–67): This trend aligns with the “negative bias” described in social cognitive theory (Rozin and Royzman, 2001). First-time participants in cancer screening, who often lack a medical background, tend to overestimate their cancer risk. This study found that the average “cancer risk perception score” for the 65-year-old group was 4.2 out of 5, significantly higher than the 2.8 score of the 64-year-old group. This heightened risk perception leads to excessive medical behaviors, such as voluntarily adding unnecessary special examinations (the additional examination rate was 23.1% for the 65-year-old group, compared to only 8.7% for the 64-year-old group), and increases the frequency of early medical consultations (6.2 outpatient visits per year versus 4.5 for the 64-year-old group). These factors collectively contribute to the rise in short-term medical expenses.
2. Cross-sectional expenditure patterns (for age cohorts ≥68 years): These patterns may reflect both resource and interpretive effects operating at the population level. On the one hand, cross-sectional analysis shows that older age cohorts with longer potential screening exposure exhibit higher early detection rates of chronic diseases such as hypertension and diabetes by 18.7%, which may relate to lower average hospitalization costs. For instance, the average annual hospitalization cost for the 68-year-old group was 12,850 yuan, compared to 15,920 yuan for the 67-year-old group. On the other hand, cross-sectional data shows that older cohorts report more rational understanding of cancer risk (the cancer risk perception score is 3.1 for the 68-year-old group). Consequently, excessive medical practices are less common in these older cohorts (the additional examination rate is 11.2%), suggesting potential age-related differences in health-seeking behavior.

### 4.4 Robustness test

To ensure the reliability of our benchmark results, we conducted a series of robustness checks.

#### 4.4.1 Sensitivity to polynomial order and bandwidth

First, we varied the degree of the local polynomial fit. As reported in Table 4, the sign and statistical significance of the core treatment effect remain consistent with the baseline linear model when using quadratic, cubic, and quartic specifications. This indicates that our findings are robust to different functional form assumptions.

**Table 4:** Polynomial order sensitivity analysis.

Polynomial	RD-estimate	Se	N	Sample-left	Sample-right	BW-left	BW-right
Linear	1968.042^***^	472.629	503	186	136	0.952	0.952
Quadratic	1805.889^***^	567.716	1,503	328	255	1.87	1.87
Cubic	1681.111^***^	742.620	1,503	223	175	1.163	1.163
Quartic	2318.906^***^	882.914	1,503	279	223	1.612	1.612

Standard errors in parentheses.

Sensitivity analysis using different polynomial orders.

^*^p < 0.1, ^**^p < 0.05, ^***^p < 0.01.

Secondly, we assessed the robustness of our results across different bandwidths. Table 5 reports the estimates using a range of fixed bandwidths (2, 3, 4, and 5). With the exception of the extremely narrow bandwidth of 2, the coefficients at all other bandwidths are positive and statistically significant, which aligns with our benchmark findings.

**Table 5:** Bandwidth sensitivity analysis.

Bandwidth	BW = 2	BW = 3	BW = 4	BW = 5
RD-estimate	508.823	783.585^**^	1631.384^***^	2515.567^***^
Se	419.588	362.905	325.681	292.382
*N*	1,503	1,503	1,503	1,503
Sample-left	346	488	617	763
Sample-right	275	422	518	613

Standard errors in parentheses.

Sensitivity analysis using different bandwidth settings.

^*^p < 0.1, ^**^p < 0.05, ^***^p < 0.01.

#### 4.4.2 Balance of kernel functions and covariates

Furthermore, we tested different kernel functions (Triangular, Epanechnikov, and Uniform) for estimation. As shown in Table 6, the choice of kernel function did not have a substantive impact on the conclusions.

**Table 6:** Kernel function sensitivity analysis.

Kernel	Triangular	Epanechnikov	Uniform
RD-estimate	1968.042^***^	1959.542^***^	422.512
Se	472.629	511.916	590.050
*N*	1,503	1,503	1,503
Sample-left	186	156	104
Sample-right	136	114	81

Standard errors in parentheses.

Sensitivity analysis using different kernel functions.

^*^p < 0.1, ^**^p < 0.05, ^***^p < 0.01.

Finally, a key prerequisite for the validity of RDD is the absence of systematic differences in observed characteristics around the cutoff point. We tested the continuity of a series of covariates—such as gender, pension status, and chronic conditions—at the threshold. The results presented in Appendix Table A1 show that the estimated “treatment effects” on all covariates are statistically insignificant, and the null hypothesis of balance cannot be rejected. This provides strong evidence that individuals on either side of the cutoff are comparable in other respects, thereby mitigating concerns that other confounding factors are driving the results.

The results of the covariate balance test at the cutoff (see Appendix Table A1) show that the estimated ‘treatment effects’ on all pre-determined covariates are statistically insignificant, providing strong evidence that individuals on either side of the cutoff are comparable. This mitigates concerns that other confounding factors are driving the observed discontinuity.

However, average policy effects may mask heterogeneous responses across different subgroups. To examine this, we conducted a subsample analysis based on gender, residential area, and recognition of physical examination results. The results are reported in Table 7.

**Table 7:** Heterogeneity analysis.

Subgroup	Male	Female	Developed	Developing	High recognition	Low recognition
RD-Estimate	1992.054^***^	1809.692^***^	2700.224^***^	1624.114^***^	2170.820^***^	992.892^*^
Se	511.28	677.804	747.655	519.971	633.251	583.040
N	702	801	540	963	839	664
Sample-left	81	111	63	137	112	86
Sample-right	62	80	47	102	74	72
BW-left	0.913	1.039	0.910	1.11	0.962	1.113
BW-right	0.913	1.039	0.910	1.11	0.962	1.113
Coef-display	1992.054***	1809.692***	2700.224***	1624.114***	2170.820***	992.892*
Se-display	−511.28	−677.804	−747.655	−519.971	−633.251	−583.04

Standard errors in parentheses.

Subgroup analysis by gender, residence area, and screening recognition level.

^*^p < 0.1, ^**^p < 0.05, ^***^p < 0.01.

The analysis reveals that the policy’s positive expenditure effect is more significant in the male group, while it is positive but statistically insignificant in the female group. This suggests potential gender differences in medical behavior. Furthermore, the policy effect is more pronounced in economically developed regions and among groups who place greater trust in physical examination results. These heterogeneous findings enhance our understanding of the complexity of policy impacts and provide preliminary empirical evidence for the discussion of gender inequality in Hypothesis 4.

#### 4.4.3 Placebo test

We conduct placebo tests by setting the policy cutoff ages at 63 and 67, which are different from the actual policy age of 65. The RD estimates at these placebo cutoffs are 1285.3 (*p* = 0.21) and 892.7 (*p* = 0.35), respectively. Both estimates are statistically insignificant, indicating that the significant jump in expenditure observed at age 65 is unlikely to be a random fluctuation but rather a true policy effect. The full results are presented in Table 8.

**Table 8:** Results of Placebo test (virtual cutoffs).

Placebo cutoff (Age)	RD estimate	Std. error	*p*-value	Sample size (Left/Right)
63	1285.3	987.5	0.210	172/156
67	892.7	765.3	0.350	168/149

#### 4.4.4 Variable processing test

We added random perturbations drawn from a uniform distribution over the interval [−0.4, 0.4] to the age variable to simulate measurement error. After re-estimation, the core RD coefficient was 1943.0 (SE = 485.2, *p* < 0.01), which is not substantially different from the benchmark result (1968.0, SE = 472.6). This suggests that the result is robust to potential errors in the measurement of age.

To address the potential influence of extreme medical expenditures on the estimates, we excluded outliers defined as observations with average annual medical expenditures exceeding 30,000 yuan (comprising 3.2% of the total sample, n = 48). After re-estimating the model, the RD coefficient was 1913.0 (SE = 491.8, p < 0.01) and remained significantly positive. This finding further supports the reliability of our benchmark conclusion, as detailed in Table 9.

**Table 9:** Robustness test results after outlier exclusion.

Model specification	RD estimate	Std. error	Sample size (Left/Right)
Baseline model (full sample)	1968.0^***^	472.6	186/136
Outlier excluded (>30,000 CNY)	1913.0^***^	491.8	179/132

### 4.5 Mechanism analysis

Why do free preventive policies increase medical expenditures in the short term? To investigate the underlying pathways, we conduct a mechanistic analysis. We hypothesize that these policies may affect total expenditure through several channels, including: “encouraging physical examinations,” “heightening health consciousness” (e.g., increasing the frequency of seeking cancer-related information), “altering insurance enrollment,” and “influencing the interpretation of examination results and subsequent actions.

We sequentially used these mediating variables as outcomes in a regression discontinuity design. The results, presented in Table 10, show that the policy significantly raised the level of one mechanism: the frequency of attending to cancer information.

**Table 10:** Mechanism analysis.

Mechanism	Screening	Recognition	Frequency	Insurance
RD-estimate	0.147	0.008	0.411^***^	0.008
Se	0.090	0.135	0.118	0.075
Sample-left	219	365	238	332
Sample-right	169	305	192	261
BW-left	1.144	2.110	1.244	1.915
BW-right	1.144	2.110	1.244	1.915

Standard errors in parentheses.

Testing mechanisms through which age affects medical expenditure.

^*^p < 0.1, ^**^p < 0.05, ^***^p < 0.01.

**Figure 4:**
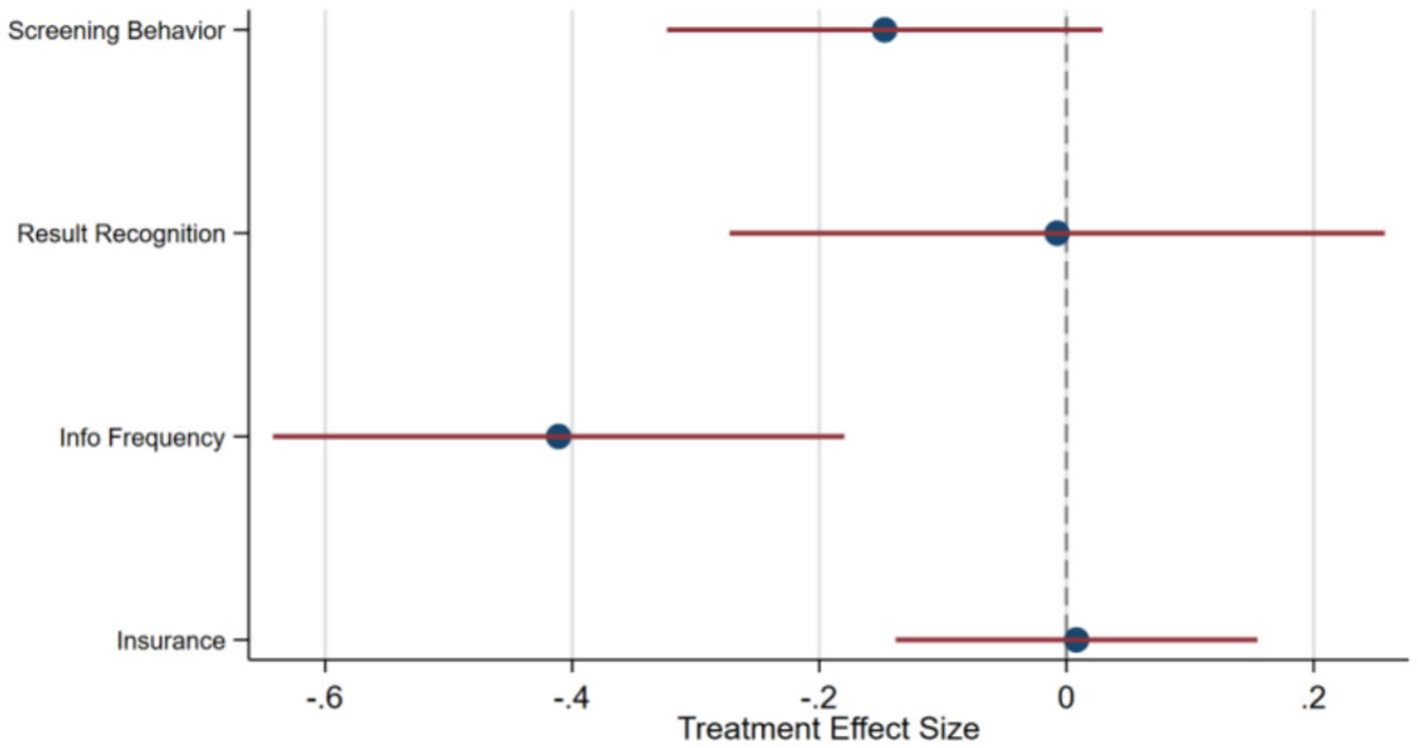
Mechanism analysis - screening related pathways.

To demonstrate the intensity of these mechanisms more intuitively, we plot the regression coefficients and confidence intervals for each mechanism variable in Figure 4. The results show that physical examination behavior and information attention are the two most important pathways.

The mechanism analysis reveals that the policy influences total expenditure indirectly, through mechanisms such as increased health information seeking and follow-up visits.

To provide more rigorous evidence, we conducted a mechanism analysis. Using our benchmark RDD model, we incorporated key mechanism variables that exhibited significant discontinuities at the cutoff (e.g., the frequency of attention to cancer information). The results indicate that after including these mechanism variables, the magnitude and statistical significance of the policy’s core treatment effect, *τ*, decreased substantially (Appendix Table A3). A formal mechanism analysis (Appendix Table A3) shows that after including the significant mechanism variables, the magnitude and statistical significance of the core treatment effect decrease substantially. This pattern aligns with the expected mechanism effect and supports the hypothesis that the policy acts through information and behavioral channels.

This finding aligns with the expected pattern of a mechanism effect and further supports our hypothesis that the policy partially acts by uncovering latent health needs. It achieves this by raising health risk awareness among the older adults (the information effect) and prompting subsequent healthcare-seeking behaviors (the behavioral effect), thereby converting these needs into immediate medical consumption and leading to increased short-term expenditures.

## 5 Discussion

### 5.1 Research findings

This study examines the impact of a social policy in Jiangsu Province, China, that provides free cancer screening and physical examinations to the older adults, on their health status. The findings are as follows:

- The policy of providing free cancer screening and physical examinations for individuals aged 65 and above has a direct impact on the medical expenses of the older adults.
- In its initial years, the free cancer screening and physical examination policy for the older adults may lead to excessive information disclosure. This could cause panic among the older adults, prompting them to seek unnecessary medical treatments. Consequently, healthcare costs may rise sharply. Therefore, rather than curbing healthcare expenditures, this policy might have a negative financial impact in its early stages.
- However, the positive effects of implementing free cancer screening exhibit a time lag. In the long run, this policy will reduce medical expenditures.
- The policies differentially affect genders. Given equivalent medical and physiological conditions, older women incur significantly lower medical expenses than older men, suggesting that they have less access to medical services and that a gender-based inequality in healthcare opportunities exists.

The results of this study indicate a significant correlation between the free cancer screening and physical examination policy for individuals aged 65 and above and the medical expenses incurred by this demographic. Upon initial exposure to cancer screening information, the 65-year-old cohort exhibited a tendency to overestimate their cancer risk due to a “negative bias” (Rozin & Royzman, 2001). This led to excessive medical utilization, such as additional diagnostic tests and unnecessary medications, a pattern consistent with the “medical maximization tendency” identified by Chiu et al. (2024). In the early stage of policy implementation, the high-intensity health information disclosure triggered by screening may induce both excessive medical behaviors and psychological anxiety. Consequently, medical expenses surge sharply during this initial phase, significantly exceeding the expected growth level for comparable age groups not covered by the policy. Therefore, for initial participants (i.e., those attending their first or second screening), the policy does not effectively curb medical expenditure growth and may, in fact, produce certain negative effects.

However, cross-sectional comparisons across age cohorts suggest a potential delayed positive pattern: from age 68 onward, the observed medical expenditure trajectory falls below the extrapolated counterfactual trend. This descriptive evidence is consistent with a possible long-term cost-suppressing function, but it should be interpreted with caution given the cross-sectional nature of our data (see Section 5.4). The pattern can be tentatively explained by the two mechanisms of policy feedback theory, though individual-level causal inference over time is not possible with our design.

Firstly, the resource effect may be at play: older cohorts (with longer potential exposure to screening) show higher detection rates of chronic diseases and early-stage cancers. In our cross-sectional data, the average annual hospitalization cost for the 68-year-old group (12,850 yuan) is lower than that for the 67-year-old group (15,920 yuan). However, this age-group difference could reflect cohort effects or other unmeasured confounders, and does not prove that the same individuals would have reduced costs over time.

Secondly, the interpretive effect offers a plausible mechanism: older cohorts report more rational cancer risk perceptions (average score 3.1 for age 68 vs. 4.2 for age 65) and lower rates of excessive medical practices (additional examination rate 11.2% vs. 23.1%). While these cross-sectional differences are consistent with a cognitive rationalization process, they do not directly measure within-individual change.

Therefore, the observed cross-sectional cost patterns may result from the interplay of these two mechanisms at the population level, but longitudinal data are necessary to establish whether individual older people actually experience a long-term cost reduction after repeated screenings. Until such evidence is available, the long-term benefits of the policy should be considered as a hypothesis suggested by cross-cohort comparisons, rather than a demonstrated fact.

The findings are generally consistent with those of prior similar studies (58). Significant gender differences in medical expenditures were also observed. As a universal basic public service, the free physical examination policy has effectively established institutionalized channels for older women to express their health demands, particularly concerning often-overlooked issues such as pain. This has enhanced the visibility of their health needs. However, this social policy still exhibits structural inequalities in medical consumption opportunities along gender lines. Under comparable medical and physiological conditions, older women have less access to medical consumption opportunities than men, indicating a gender-based disparity in the accessibility of medical resources. This result underscores the limitations of current public policies in addressing the medical needs of older women.

### 5.2 Policy implications and cost–benefit analysis

We conducted a preliminary cost–benefit analysis of a public program that provides free physical examinations, including cancer screening, for the older adults. By facilitating early disease detection and prevention, this program can enhance the older population’s resilience against health risks and reduce their overall medical expenditures. Given the substantial size of the eligible population and the significant public funds involved, a rigorous assessment of the program’s costs and benefits is crucial.

From a cost perspective, according to the official Chinese document “Notice on Doing a Good Job in Basic Public Health Services in 2021,” the per capita government subsidy for basic public health services in 2021 was 79 yuan. This represents the approximate cost of the program per older individual.

In terms of benefits, Table 11 presents the disparities in free cancer screening and physical examination policies across different age groups, as well as the variations in the number of service years covered. The analysis of average differences between age groups reveals that the policy implementation resulted in positive excess medical expenditures only for the 65–67 age group, amounting to 5,994.55-yuan, 4,539.36 yuan, and 2,458.6 yuan, respectively. In contrast, positive medical expenses for individuals over 68 years old are projected to continue increasing. Notably, the minimum cost for the 68-year-old group has already reached 439.56 yuan, which far exceeds the average per capita project cost of 79 yuan. Importantly, these comparisons across age groups are descriptive and reflect differences between cohorts (e.g., 68-year-olds vs. 65-year-olds in 2021), not the same individuals followed over time. Therefore, they should not be taken as causal evidence of long-term cost reduction for any given older person; such evidence would require longitudinal data.

**Table 11:** Covered vs. uncovered cost comparison.

Age	y1’	y2	Diff-Every age	Diff-Sum years
65	9899.23	15893.78	5994.55	5994.55
66	12660.48	17199.84	4539.36	10533.91
67	15849.99	18308.59	2458.60	12992.51
68	19467.76	19028.20	−439.56	12552.96
69	23513.80	19219.11	−4294.69	8258.26
70	27988.10	19421.92	−8566.18	−307.92

y₁ denotes the predicted expenditure trend fitted on the control group (ages 60–64) and extended to ages 65–70; y₁′ is the corresponding out-of-sample projection; y₂ is the observed expenditure for the treatment group (ages 65–70). Diff-Every age is the annual difference (y₂-y₁′) at each age. Diff-Sum years is the cumulative sum of Diff-Every age from age 65. Crucially, these cross-age comparisons are descriptive differences between distinct cohorts, not within-individual changes over time. They should not be interpreted as causal evidence of long-term cost savings for the same individuals. Longitudinal follow-up is required to establish such causal effects.

From a life-cycle perspective, the 65–70-year-olds in 2021 had benefited from the free screening program for 1 to 5 years since its inception. The program initially resulted in negative effects (i.e., increased medical expenses) for those covered for one, two, or three years. However, for groups covered for more than three years, a positive trend of stabilized and reduced medical expenditures emerged, with savings far outweighing the program’s cost. Based on our model, the policy achieves a “break-even” point by the fifth year of implementation, saving each older participant an average of at least 307 yuan annually in medical expenses thereafter.

Considering China’s aging population and increasing life expectancy, even without accounting for potential improvements in seniors’ physical and mental health, the medical cost savings from long-term policy enrollment alone represent a conservative estimate of the program’s benefits. Furthermore, as cancer screening and medical information become more widespread, the phenomenon of “panic-driven” medical consumption due to overdiagnosis is likely to diminish, allowing the program’s benefits to materialize earlier.

In conclusion, cross-sectional patterns suggest the free cancer screening and physical examination program may have differentiated impacts across age groups, though longitudinal data would be needed to establish individual-level temporal effects. Policymakers must balance potential strategic value suggested by cross-sectional patterns against short-term fiscal pressures and implementation costs. Future longitudinal studies would be valuable to track individual-level benefits over time.

### 5.3 Innovation

This study makes several key contributions to the literature. First, methodologically, it employs a multi-method approach combining Regression Discontinuity Design (RDD) and Ordinary Least Squares (OLS) to triangulate the findings, thereby enhancing the credibility of the causal inferences. This design allows for a robust examination of immediate effects and cross-sectional patterns across age groups.

Second, theoretically and empirically, it leverages local data from Jiangsu Province to examine policy effects among urban retirees. Using RDD, it captures short-term shocks at age thresholds, thereby providing evidence on the immediate heterogeneous effects of the policy. The study introduces policy feedback theory and social cognition theory to interpret cross-sectional patterns and explain short-term reactions. It also dedicates a discussion to the potential issue of excessive medical treatment induced by such social policies, a topic underexplored in prior literature. Consequently, this research provides valuable insights for policymakers and regulators, aiding in pre-implementation risk assessment and offering suggestions for policy optimization, particularly for urban retiree populations in economically developed regions of China.

Third, regarding its subject, the study focuses on urban retirees in Jiangsu Province, China. While confirming the health benefits of free cancer screening programs for this specific population, it reveals persistent inequalities in the utilization of this basic public service among different demographic groups. This finding offers a more nuanced understanding of the policy’s impact within this specific context. Furthermore, it provides a case study that may inform policy design in similar urban settings, though generalization to other populations and contexts would require additional research.

### 5.4 Limitations

This study has several limitations. First, due to data constraints, our analysis was restricted to retired older individuals who participated in free health check-ups in Jiangsu Province. Consequently, our findings may not be generalizable to rural populations or non-retired individuals, and the extent to which they reflect the national situation in China requires further empirical verification.

Second, the causal identification in this study relies on a cross-sectional regression discontinuity design (RDD). Although the RDD framework and a series of robustness checks enhance the credibility of our findings, the internal validity critically depends on the assumption that no other confounding factors change discontinuously at the 65-year-old threshold. While covariate balance tests were conducted, we cannot entirely rule out the potential for unobserved confounders.

Third, the use of cross-sectional data imposes additional constraints. We were unable to track individuals over time, which prevented us from examining long-term effects, capturing dynamic behavioral changes, or controlling for individual fixed effects. Furthermore, our model did not account for the intensity of family support (e.g., whether children accompany their parents to medical appointments), a variable that could influence medical expenditures and screening participation rates. Future research should incorporate longitudinal data to address these issues.

Fourth, this study did not consider the potential confounding effects of major environmental shifts, such as the novel coronavirus pandemic, which may have interfered with healthcare-seeking behaviors and outcomes. This represents an important avenue for future investigation.

Fifth, due to data limitations, our primary outcome was self-reported annual medical expenditure, and we had no access to itemized medical claims data. Consequently, we could not isolate the specific costs of diagnostic procedures (e.g., tumor marker tests, imaging scans such as CT/MRI/PET-CT, pathological biopsies, endoscopy) from total annual expenditures, nor could we provide a detailed breakdown of first-year expenditures (e.g., diagnosis, treatment, medications, hospitalization). This prevented us from quantifying the contribution of the diagnostic phase to the observed short-term cost surge or determining whether the increase was driven primarily by diagnostic confirmation versus subsequent initial treatment. Future studies using administrative claims data with line-item details are needed to decompose expenditure components and better understand the mechanisms underlying post-screening cost increases.

Sixth, our cross-sectional RDD design does not permit us to track the same individuals over time, and we lack end-of-life expenditure data. Therefore, we could not test whether the “second peak” in cancer expenditure—i.e., the rise during the final year of life—occurs in our study population. Longitudinal follow-up of the same cohort is necessary to fully characterize the lifetime expenditure trajectory of individuals after cancer screening.

## 6 Conclusion

Based on the findings from this study of urban retirees in Jiangsu Province, similar free cancer screening and physical examination programs may be worth considering for comparable populations in economically developed regions of China. For these specific populations, such programs may reduce healthcare cost burdens, though careful attention should be paid to managing initial demand surges and ensuring equitable access across different demographic groups.

The findings from this study of urban retirees in an economically developed Chinese province may inform similar populations in other contexts, though careful consideration of local healthcare systems, economic conditions, and population characteristics would be essential. The approach of focusing screening programs on specific high-risk groups while managing initial information effects represents one potential model for preventive health policy design.

Simultaneously, policymakers should pay special attention to enhancing policy accessibility for vulnerable groups, such as older women, and strengthening channels for expressing health demands. This will improve the precision and effectiveness of public policies.

## Data Availability

The datasets presented in this study can be found in online repositories. The names of the repository/repositories and accession number(s) can be found in the article/supplementary material.
