# Supplementary figures and images for: Cancer screening as a double-edged sword: short-term cost surge and cross-sectional expenditure patterns among urban retirees in Jiangsu, China
Source: Front Public Health. 2026 Jun 25;14:1822162. doi: 10.3389/fpubh.2026.1822162 (PMC13346067; doi:10.3389/fpubh.2026.1822162)

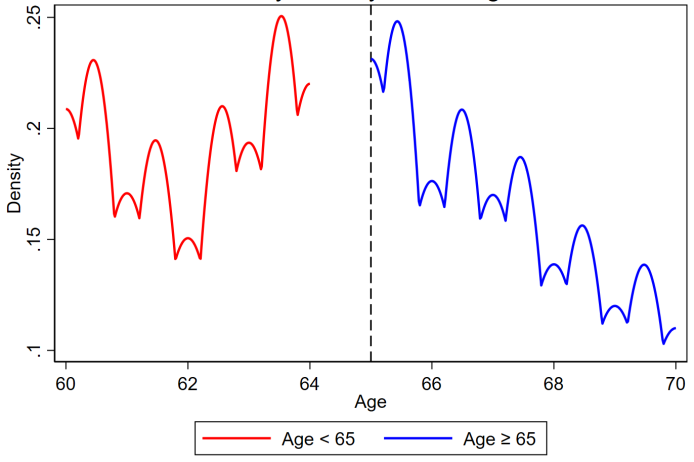

Supplement: Supplementary file 1 [file Image_1.tif]
