# Supplementary material for: Cancer screening as a double-edged sword: short-term cost surge and cross-sectional expenditure patterns among urban retirees in Jiangsu, China
Source: Front Public Health. 2026 Jun 25;14:1822162. doi: 10.3389/fpubh.2026.1822162 (PMC13346067; doi:10.3389/fpubh.2026.1822162)
Supplement: Supplementary file 2 [file Supplementary_file_1.pdf]

10. Supporting Information

Figure A2 Regression Discontinuity Plot

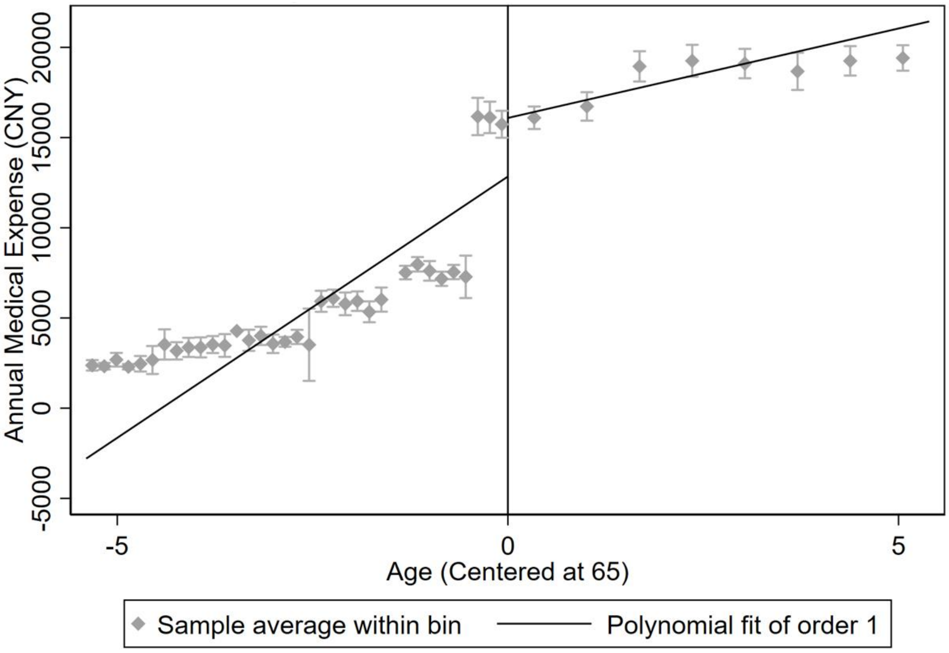

Note: Regression discontinuity plot with 95% confidence intervals. Circles represent mean annual medical expenditure within 1- year age bins. The solid lines are fitted values from a second- order global polynomial regression estimated separately on either side of the eligibility cutoff (age 65, vertical dashed line).

Table A1 Covariate Balance Test at Cutoff (Appendix)

Variable	Coefficient	SE	t	P	N
Gender	0.0996	0.0781	1.276	0.2019	615
Pension Type	0.0136	0.1569	0.087	0.9308	500
Cancer Screening Awareness	0.0489	0.0547	0.895	0.371	500
Chronic Diseases	-0.1153	0.0907	-1.271	0.2038	505
Self-rated Health	-0.0659	0.0705	-0.935	0.3497	535
Residential Status	0.166	0.0894	1.857	0.0633	789
Cancer Info Frequency	-0.4822	0.1376	-3.503	0.0005	396
Cancer Info Attention	0.0769	0.1156	0.665	0.5059	711
CSPS Score	-0.0337	0.082	-0.412	0.6806	627
Commercial Insurance	0.0026	0.078	0.034	0.9732	637

Recognition of Results	0.0052	0.138	0.038	0.97	670
Basic Insurance	0.1139	0.0804	1.416	0.1567	597
Residence Area	0.1213	0.0756	1.604	0.1086	547

TableA2 Fuzzy RDD

Coefficient	SE	P	N	R2
7120.5	480.300	0.000	1503	0.245

TableA3 Pathway Analysis

Model	RD-Coefficient	Attenuation	N
Baseline Model (without Mechanism Variables)	1968	Reference Group	1503
Frequency-Information	1724	12.40%	1503
Recognition-Results	1661	15.60%	1503
